# The lesion correlates of impaired content word fluency during spoken discourse in aphasia

**DOI:** 10.1093/braincomms/fcag071

**Published:** 2026-03-13

**Authors:** Reem S W Alyahya, Junhua Ding, Erica L Middleton, Daniel Mirman

**Affiliations:** Division of Speech and Language Therapy, Department of Allied Health, School of Health and Medical Sciences, City St George’s, University of London, London EC1V 0HB, UK; Cochlear Centre, King Fahad Medical City, Riyadh 11525, Saudi Arabia; State Key Laboratory of Cognitive Science and Mental Health, Institute of Psychology, Chinese Academy of Sciences, Beijing 100101, China; Moss Rehabilitation Research Institute, Elkins Park, PA 19027, USA; Department of Psychology, University of Edinburgh, Edinburgh EH8 9JZ, UK

**Keywords:** aphasia, discourse, content word fluency, CIU, lesion-symptom mapping

## Abstract

Human conversations are strongly dependent on the production of informative and fluent connected speech. In people with aphasia, this skill is affected to varying degrees, which can negatively impact social communication and quality of life. However, available coding systems to assess connected speech production based on discourse tasks are labour-intensive and very time-consuming, limiting their utilization in clinical and research contexts. In this study, we investigated and further validated a recently developed, accurate, time-efficient and clinically applicable measure of content word fluency (CWF) during spoken discourse in a large, unselected sample of 76 participants with chronic aphasia following left hemisphere stroke. We report the first identification of lesion correlates of impaired content word production in spoken discourse using state-of-the-art lesion-symptom mapping methods, including voxel-wise disconnection, multivariate lesion-symptom mapping and tract-wise analyses. Discourse responses elicited using composite picture description were analysed using (i) CWF to assess content word production in spoken discourse using a pre-specified checklist without transcription or quantitative analysis and (ii) ‘correct information unit’ (CIU) following the standard transcription and quantitative analysis protocol of discourse samples. We showed a significant, strong positive correlation between CWF scores and the number of CIUs. Item Response Theory analysis revealed that the one-parameter logistic model best fits the data, indicating that items on the CWF checklist are homogeneously measuring a single underlying construct. Moreover, the items on the checklist were found to have distributed difficulties in the sample over a large functional range, indicating that the CWF approach is sensitive to variations in performance across a broad spectrum of aphasia severity. The neuroimaging findings indicated overlapping lesion correlates between CWF and CIU in the left frontal and parietal regions, and anterior dorsal white matter pathways, specifically the middle frontal gyrus, inferior parietal lobe, frontal aslant tract and superior longitudinal fasciculus. These results reveal strong convergence between CWF and CIU, and they provide behavioural, neurological and psychometric validation of the CWF approach, an efficient tool for assessing communication deficits in people with aphasia in both clinical and research settings. These insights have potential clinical implications, from improving targeted rehabilitation strategies to predicting recovery outcomes.

## Teaser

Alyahya *et al*. provided empirical validation for a recently developed time-efficient and clinically applicable approach to measuring content word fluency during spoken discourse (CWF approach). The authors discovered lesion correlates in the left frontal and parietal regions, and the anterior dorsal white matter pathways, related to impaired content word fluency in aphasia.

## Graphical Abstract

**Graphical Abstract:**
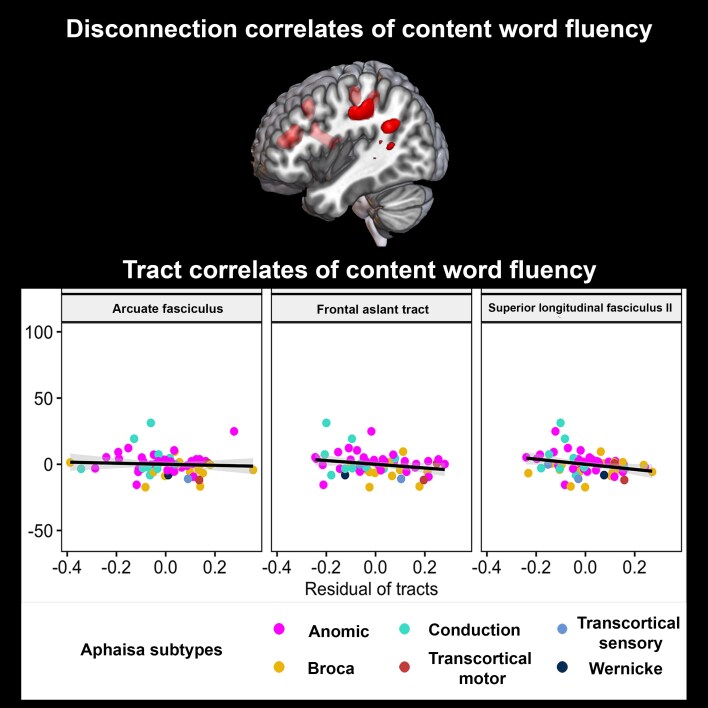

## Introduction

People with aphasia (acquired language and communication impairments) due to stroke or other neurological disorders often have difficulty producing discourse and informative connected speech. This can lead to reduced participation in conversations, impacting their social engagement and quality of life.^1^ While current cognitive models and neuroimaging research focus on single-word and sentence processing (e.g. phonology, semantics and syntactic complexity), there is a notable lack of research on effective and efficient communication at the discourse level. Understanding the neural underpinnings of spoken discourse is crucial for theoretical and clinical reasons, but it has been relatively underexplored in the literature.^2^

Discourse is language used beyond sentences and phrases to communicate messages in a meaningful and interactive way.^3^ The cornerstone of informativeness in spoken discourse is the production of content words. Correct information unit (CIU) is a well-established approach, which uses a standardized scoring method to quantify the informativeness of spoken discourse.^4^ This approach has strong psychometric properties of reliability and construct validity, high diagnostic sensitivity, and good ecological validity.^4,5^ A recent lesion-symptom mapping (LSM) study of left-hemisphere stroke aphasia revealed that impaired production of relevant information (number of CIU: #CIU) was associated with damage to anterior dorsal white matter pathways, specifically the frontal aslant tract, arcuate fasciculus and superior longitudinal fasciculus.^6^ These results contribute to the limited literature on the neural underpinnings of informativeness in spoken discourse. This body of research found an association between lesions to the left inferior frontal gyrus, frontal operculum cortex and the frontal aslant tract with impaired production of content words, informativeness and global coherence in spoken discourse.^7-10^

The gold standard approach to assessing spoken discourse involves the laborious process of collecting, transcribing and analysing discourse samples. For example, CIU coding requires counting the relevant, intelligible, accurate and reliable words from each transcribed discourse sample.^4^ This is very effortful and time-consuming. Transcription and analysis of a 1-min discourse sample can take up to an hour, depending on aphasia severity and analytic demands.^11^ This could contribute to the limited aphasiological and neuroimaging studies on discourse production. To overcome this challenge, a recent transcription-less approach to discourse analysis has been developed and validated across healthy adults and people with aphasia (PWA).^12^ Specifically, content word fluency (CWF) is an efficient approach to quantify the fluency of content words in spoken discourse without the need to collect and transcribe discourse samples.^12^ This approach is stimulus-dependent, and it involves deriving checklists of target items from the discourse responses of an independent group of neurotypical controls. CWF checklist has been developed for the ‘Cookie Theft’ composite picture description^13^ and validated across a large group of PWA.^12^ The convergent validity of this approach has been examined by directly contrasting performance using this novel approach to that derived from traditional transcription and quantitative analyses of spoken discourse samples (i.e. correlating scores on CWF to #CIUs). Specifically, very high correlations have been reported between CWF and #CIUs among neurotypical controls and PWA.^12^ A recent study provided evidence that the CWF approach can be applied successfully and efficiently in real time during clinical examination of PWA.^14^ However, it remains unclear whether impaired CWF reflects deficits in word fluency and/or discourse informativeness during spoken discourse.

The overarching aim of the current study is to further validate the CWF approach and enhance our understanding of deficits associated with impaired fluency of content words during discourse production. We accomplished this in three steps. First, we replicated the findings of Alyahya *et al*.^12^ using an independent dataset of discourse responses produced by a large cohort of PWA. Second, we explored the sufficiency of the current number of words on the CWF checklist using item-based analyses. The third step was to identify the crucial brain regions associated with CWF during spoken discourse and determine if these are similar or different to the neural correlates associated with #CIU.

## Materials and methods

Behavioural data collection and sharing were approved by the Institutional Review Board at the Einstein Healthcare Network. The neuroimaging data were collected at the University of Pennsylvania School of Medicine and approved by its Institutional Review Board. Analysis of de-identified data was approved by the University of Edinburgh PPLS Research Ethics Committee.

### Participants

The dataset consisted of 76 participants (35 females) who had a left-hemisphere stroke that resulted in aphasia. Participants were at least 3 months post-stroke onset (to capture more severe subtypes of aphasia), but without selection based on aphasia type or severity. The same sample was included in a previous study.^6^ All participants were right-handed and native English speakers. To participate in this study, participants must generate a verbal response (i.e. to name one picture on a picture naming test). These participants were recruited from the Moss Rehabilitation Research Registry. Exclusion criteria involved neurodegenerative diseases, dementia, psychosis, developmental disability, brain tumours, encephalitis, significant sensory disturbances (profound deafness or blindness in both eyes) or seizures pre-existing their stroke. Participants’ language abilities were assessed using the Western Aphasia Battery (WAB).^15^ The standard WAB protocol was used to classify Aphasia subtypes. Subtests of comprehension, spontaneous speech, repetition and naming were used to calculate the aphasia quotient as a measure of aphasia severity. Naming, comprehension and repetition abilities were further assessed using the Philadelphia naming test (PNT),^16^ the Camel and Cactus test of semantic comprehension,^17^ a word repetition test based on the PNT^18^ and a non-word repetition test.^19^ Table 1 presents the demographic and neuropsychological information for the participants.

**Table 1:** Demographic and neuropsychological characteristics of the participants (*N* = 76)

Variable		Mean	Median	IQR	Range
Demographics	Age (years)	58	58	50–68	30–78
	Education (years)	15	14	12–18	10–21
	Post-onset (months)	54	23	8–78	4–266
	Lesion size (cc)	99	77	48–133	5–376
WAB	AQ (max 100)	79	82	70–90	47–99
	Fluency (max 10)	7	8	5–9	2–10
	Repetition (max 10)	8	8	7–9	2–10
	Comprehension (max 10)	9	9	8–10	6–10
	Aphasia severity	Severe: 3 Moderate: 25 Mild: 41 Latent aphasia: 6			
	Aphasia classification	Broca’s: 17 Wernicke’s: 2 Anomic: 41 Conduction: 10 Transcortical sensory: 2 Transcortical motor: 3			
Language battery	Philadelphia naming test (% accurate)	73	78	63–86	15–78
	Philadelphia repetition test (% accurate)	88	94	85–97	19–100
	Camel and cactus test (% accurate)	76	78	71–83	31–94
	Non-word repetition (% accurate)	50	51	31–70	0–95
Discourse task	Number of words	83	71	50–106	7–278
	#CIU	47	41	26–59	3–150
	CWF score	16	16	11–22	1–49

### Discourse task (stimuli and coding)

Spoken discourse samples were elicited using the ‘Cookie Theft’ composite picture description from the Boston Diagnostic Aphasia Examination(BDAE).^13^ For the CWF approach to be used in clinical practice, it was important to sample across the whole aphasia spectrum and include a range of severity levels, as this would reflect the standard clinical profiles of PWA. Therefore, no minimum number of elicited words was required.

Discourse responses were audio-recorded. Four trained technicians transcribed and marked the timing of the discourse responses and scored the #CIU.^4^ #CIU consists of all intelligible, accurate and informative words that are relevant to the eliciting stimulus, based on the rules of Nicholas *et al*.^4^ Discourse samples produced by six participants were coded by three raters to test inter-rater reliability. Pairwise agreement for words was 99%, and #CIU inter-rater reliability was 95%. Additionally, discourse samples from five participants were scored twice by a fourth rater to test intra-rater reliability. Agreement of words was 99%, and intra-rater reliability for #CIU was 93%. These findings indicate excellent intra-rater and inter-rater reliability.

The discourse samples were also analysed to obtain CWF scores for each participant, using the same approach used in Alyahya *et al*.^12^ Target words on the CWF checklist of the ‘Cookie Theft’ picture description consist of 22 words.^12^ These target words were extracted from the ‘Cookie Theft’ composite picture samples produced by an independent group of neurotypical controls, and they reflect content words that were produced consistently and commonly by the majority of neurotypical participants (i.e. ≥75%) irrespective of the word class (for more details, see Alyahya *et al*.^12^). The coding involved assigning one point every time the participant produced an item from the checklist, including any inflected form of verbs or nouns, any synonyms from the checklist and when the target words were used again in a subsequent phrase, but excluding immediate repetition or perseverations of the same word. A psychometric evaluation of the CWF approach indicated a significant excellent test-retest reliability (ICC = 0.94) and significant excellent inter-rater reliability (ICC = 0.991) during descriptive discourse when 25% of the discourse samples produced by PWA were randomly coded by another rater.^14^ Discourse information is provided in Table 1.

### Lesion data

Lesion masks were available for 63 participants (45 MRI and 18 CT). The masks were manually drawn according to an established protocol^20^ and were used in our previous work.^21^ A trained technician manually segmented lesions on each participant’s T_1_-weighted structural image on the MRI scans, and the accuracy of these lesions was reviewed by an experienced neurologist. An automated symmetric diffeomorphic registration algorithm was used to register each participant’s brain image to the Montreal Neurological Institute space Colin27 template.^22^ This image transformation solution was then applied to the lesion mask to register it to the same Colin27 template. On the other hand, an experienced neurologist drew the lesions on the CT scans directly onto the Colin27 template after rotating it based on pitch only to match the approximate slice plane of the participant’s scan.

### Statistical analyses

Initially, we explored the relationship between aphasia severity (measured using WAB AQ) and performance on the CWF approach and #CIU using Pearson’s correlation analyses. We also compared the performance of people with anomic aphasia versus those with Broca’s aphasia on CWF scores and #CIU using independent *t*-tests.

#### Behavioural analysis

To further validate the CWF approach, we replicated the findings of Alyahya *et al*.^12^ using their validation method on an independent dataset of discourse responses produced by PWA. Specifically, Pearson’s correlation coefficient was calculated between CWF scores and #CIU. We also performed Item Response Theory (IRT) analysis of the checklist’s target words. The analysis was conducted through R package ‘mirt’ (https://cran.r-project.org/web/packages/mirt/index.html) and ‘ggmirt’ (https://github.com/masurp/ggmirt). There could be one to three parameters for the model, and model fit was compared with a chi-square statistic. If no significant difference existed, we used the simpler model. The M2 statistic was used to test whether the model was different from the data or not.^23^ After building the model, item coefficients were extracted to reflect their difficulty.

To test the degree to which impaired CWF during spoken discourse was attributable to the psycholinguistic features of the word, we conducted a simultaneous multiple regression model. We identified the psycholinguistic variables for each word on the CWF checklist (Table 2) as follows: (i) word class (noun versus verb) determined using the Merriam-Webster dictionary; (ii) length (number of phonemes); (iii) frequency values (combined written and spoken lemma frequency counts per million words, obtained from the British National Corpus^24^); and (iv) imageability ratings (drawn from the MRC Psycholinguistic Database^25^). In the regression model, ordinary least squares method was used, where CWF scores across participants were summed and entered as the dependent variable, and the psycholinguistic variables were used as independent variables.

**Table 2:** The psycholinguistic variables of target words on the CWF checklist of the Cookie Theft picture description

Variable	Mean or ratio	SD	Range
Length (# phonemes)	3.65	1.27	2–7
Frequency	3.10	1.16	0.77–4.56
Imageability	512.41	133.74	230–638
Words class (nouns: verbs)	15: 7		

#### Lesion-symptom mapping analysis

To investigate the lesion correlates associated with impaired production of fluent content words during spoken discourse, we performed a multivariate voxel-wise LSM using the SCCAN algorithm implemented in the R package LESYMAP.^26^ Participants’ CWF scores were the dependent variable, and total lesion size was regressed out from each CWF score before LSM was performed. The analysis only included voxels that were damaged in at least 10% of the participants. SCCAN is an optimization algorithm that finds an optimally ‘sparse’ set of weights that maximizes the association between voxel lesion values and behavioural scores. Four-fold cross-validation (CV) using the correlation between expected and actual CWF scores was used to evaluate the multivariate solution. If a model was statistically significant (*P* < 0.05), we display voxels with negative weights, because they are interpretable in predicting symptom severity (i.e. lesion in that voxel is associated with poorer performance on the CWF approach). We described regions within the neuroimaging maps with reference to the AAL3 grey matter atlas^27^ and HCP1065 white matter atlas.^28^ To investigate the lesion correlates associated with impaired CIU, we performed a similar multivariate voxel-wise LSM but using #CIU as the dependent variable. For a direct comparison between CWF and CIU results, the sparseness was always set as −0.3.

#### Disconnection lesion-symptom mapping analysis

Voxel-wise disconnection LSM is a new approach used to investigate lesion-symptom associations that result from damage to structural white matter pathways, even for brain regions that are spared after stroke.^29,30^ We generated disconnection maps using the ‘lesion quantification toolkit’.^31^ We calculated the percentage of voxel-wise disconnection by dividing each voxel’s number of disconnected streamlines by the total number of streamlines that pass through this voxel in the template. These disconnection percentages were binarized at a threshold of 50%.^29^ We used this approach to be consistent with the voxel-wise lesion LSM, which also binarized each voxel into lesioned versus non-lesioned. Multivariate SCCAN disconnection LSM was performed on CWF scores and on #CIU equivalent to the LSM analyses described above (damaged in at least 10% of participants, while controlling for lesion size).

#### Tract-based lesion-symptom mapping analysis

Dorsal white matter tracts are critical for #CIU.^6^ Therefore, we conducted a tract-based LSM to compare the neural basis of CWF and CIU. Eight language-relevant tracts were selected: frontal aslant tract (FAT), arcuate fasciculus (AF), inferior fronto-occipital fasciculus (IFOF), inferior longitudinal fasciculus (ILF), uncinate fasciculus (UF) and three segments of superior longitudinal fasciculus (SLF).^32^ The tracts’ atlas was overlaid on subjects’ lesion masks to generate lesion percentage values (voxel number of lesion mask ∩ tract atlas/voxel number of tract atlas). Partial correlations were calculated between tracts’ lesion percentage and CWF/#CIU while controlling for total lesion size.

#### Predictive validity comparison

To compare the lesion correlates associated with CWF and #CIU directly, we carried out predictive validity comparison (PVC), a method that establishes a statistical criterion for determining whether two distinct lesion-behaviour maps have higher predictive accuracy than a single map for both symptom scores.^33^ The LSM analysis was performed using the same parameters as described above. Akaike information criterion (AIC) was used to measure model fit. Total predictive accuracy was summarized using the total AIC across all subjects and both behaviours (CWF and #CIU). AIC difference > 100 indicates significantly different patterns.

## Results

As expected, there were significant, strong positive correlations between aphasia severity and both CWF (r = 0.49, *P* < 0.0001) and #CIU (*r* = 0.53, *P* < 0.0001). Moreover, there were significant differences between people with anomic aphasia and those with Broca’s aphasia in the production of CWF (*t* = 5.1(56), *P* < 0.0001) and #CIU (*t* = 7.4(56), *P* < 0.0001) during spoken discourse. These findings indicate that people with more severe and those with non-fluent types of aphasia produced fewer content words during spoken discourse.

### Further validation of the content word fluency approach

**Figure 1:**
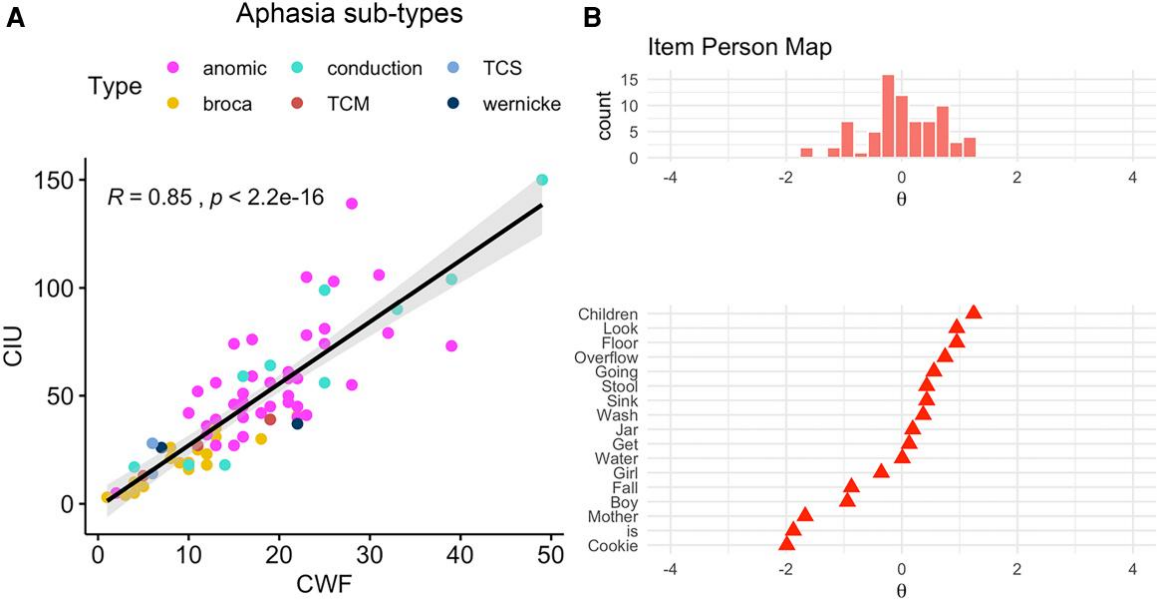
**Validation of CWF approach.** (**A**) Scatterplots illustrating the correlations between scores on the content word fluency (CWF) approach (*x*-axis) and correct information unit (#CIU) (*y*-axis) during spoken discourse in stroke aphasia (*r* = 0.85, *P* < 0.001). Each data point represents a subject (*N* = 75), including anomic aphasia (*n* = 41), Broca’s aphasia (*n* = 17), conduction aphasia (*n* = 10), transcortical motor aphasia (*n* = 3), transcortical sensory aphasia (*n* = 2) and Wernicke’s aphasia (*n* = 2). (**B**) Aligned item-person plots for Item Response Theory analysis results demonstrating that a one-parameter logistic model was the best fit (*M*^2^[135] = 162, *P* = 0.06; 1 versus 2 parameters: *x*^2^[16] = 20, *P* = 0.21; 1 versus 3 parameters: *x*^2^[33] = 26, *P* = 0.79). Plots illustrating subject counts for each checklist item (*x*-axis) and latent ability/item difficulty (*y*-axis). Each data point represents an item from the CWF checklist (*n* = 17). TCM = transcortical motor; TCS = transcortical sensory.

There was a significant, strong positive correlation between scores on the CWF and #CIU (*r* = 0.85, two-tailed *P* < 0.001), replicating the findings from Alyahya *et al*.^12^ in an independent dataset of PWA (Fig. 1A).

To further validate the CWF approach and to explore the sufficiency of the current number of words on the checklist, item-based analyses were carried out using multiple regression and IRT. The psycholinguistic variables of the checklist words only explained 28% of the variance in CWF, but the multiple regression model was not significant (*P* = 0.09), and none of the psycholinguistic variables were significant predictors of CWF during spoken discourse. The IRT analysis of the checklist words found that a one-parameter logistic model was the best fit (*M*^2^[135] = 162, *P* = 0.06; 1 versus 2 parameters: *x*^2^[16] = 20, *P* = 0.21; 1 versus 3 parameters: *x*^2^[33] = 26, *P* = 0.79), indicating that the probability of a correct response can be described by a single dimension—item difficulty. The items were found to have distributed difficulties (Fig. 1B, top) that covered the latent ability of all participants in the sample (Fig. 1B, bottom) with a quite large functional range. For example, the word ‘children’ is the most difficult item on the CWF checklist, in which only participants with the highest ability can correctly use it in spoken discourse. In contrast, the word ‘cookie’ is the easiest item on the CWF checklist, which even participants with low ability can correctly use in spoken discourse. These findings provide further validation of the CWF approach and suggest that the current number of words on the checklist is sufficient.

### The lesion correlates of impaired content word fluency during spoken discourse

**Figure 2:**
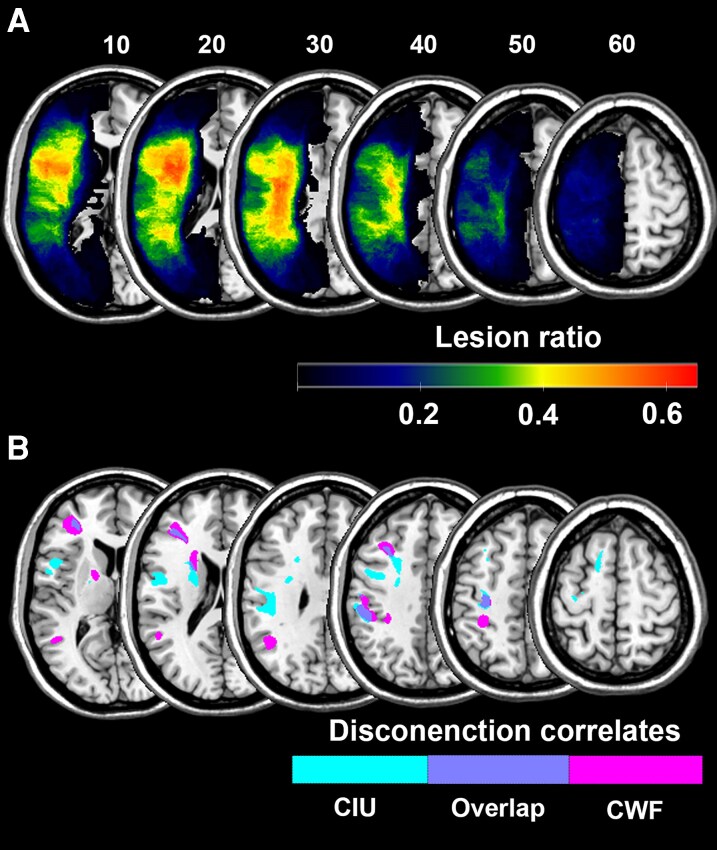
**The lesion correlates of spoken discourse measures.** (**A**) Lesion overlay map illustrating the lesion distribution across 63 participants with post-stroke aphasia. The colours represent the ratio of lesioned areas across the cohort (ranging from 0 to 1). The heatmap scale represents the number of participants with a lesion at a given location. (**B**) Lesion correlates associated with impaired content word fluency (CWF) (violet), correct information units (CIUs) (teal) and overlapping regions (blue) using disconnection LSM on participants’ lesion maps (*N* = 63) and inferential disconnection map (binarized by disconnection proportion of 0.5), respectively, to predict either CWF scores or #CIUs. Each colour represents the significant brain areas associated with impaired CWF or #CIUs. Total lesion size was regressed out from the symptom score. Values in the brain map correspond to standardized weights, indicating the contribution of each voxel to the model. Only voxels with normalized weights greater than 0.1 are shown.

The lesion overlap map is illustrated in Fig. 2A and indicates good coverage of the left middle cerebral artery territory, which is typical in post-stroke aphasia.

The LSM did not produce a statistically significant solution. Disconnection LSM produced a significant solution for CWF scores (CV correlation = 0.25, uncorrected *P* = 0.046) and #CIUs (CV correlation = 0.45, uncorrected *P* = 0.0002) (Fig. 2B and Table 3). For CWF, the identified clusters covered the left middle frontal gyrus (MFG), inferior parietal lobule (IPL) and white matter in the area of the second branch of SLF, FAT, corpus callosum (CC) and superior thalamic radiations (STR). For #CIUs, the grey matter regions were the left precentral gyrus and IPL, and the critical white matter tracts were the left CC, AF, FAT, third branch of SLF and STR. To investigate whether these cortical regions and white matter tracts are uniquely associated with one discourse approach over-and-above the other approach, we replicated the disconnection LSM on CWF scores while regressing out #CIU and vice versa. The findings did not produce significant solutions for CWF or #CIU. Moreover, PVC analysis indicated the lesion-behavioural maps for CWF and #CIU are not reliably different: AIC difference = 14, indicating that the LSM results of CWF and #CIU are adequately described as a single map rather than two distinct maps.

**Table 3:** Summary of disconnection LSM results for spoken discourse measures

	Grey matter regions	White matter tracts
CWF	IPL 19% MFG 21%	SLF2 13% CC 15% STR 11%
#CIU	IPL 13% PreCG 13%	SLF3 12% CC 14% STR 12% AF 14% FAT 20%

AF = arcuate fasciculus; CC = corpus callosum; CIU = correct information unit; CWF = content word fluency; FAT = frontal aslant tract; IPL = inferior parietal lobe; LSM = lesion symptom mapping; MFG = middle frontal gyrus; PreCG = precentral gyrus; SLF2 = second branch of superior longitudinal fasciculus; SLF3 = third branch of superior longitudinal fasciculus; STR = superior thalamic radiation.

All the regions are significant and in the left hemisphere. Only regions containing >10% of the result map are provided.

**Figure 3:**
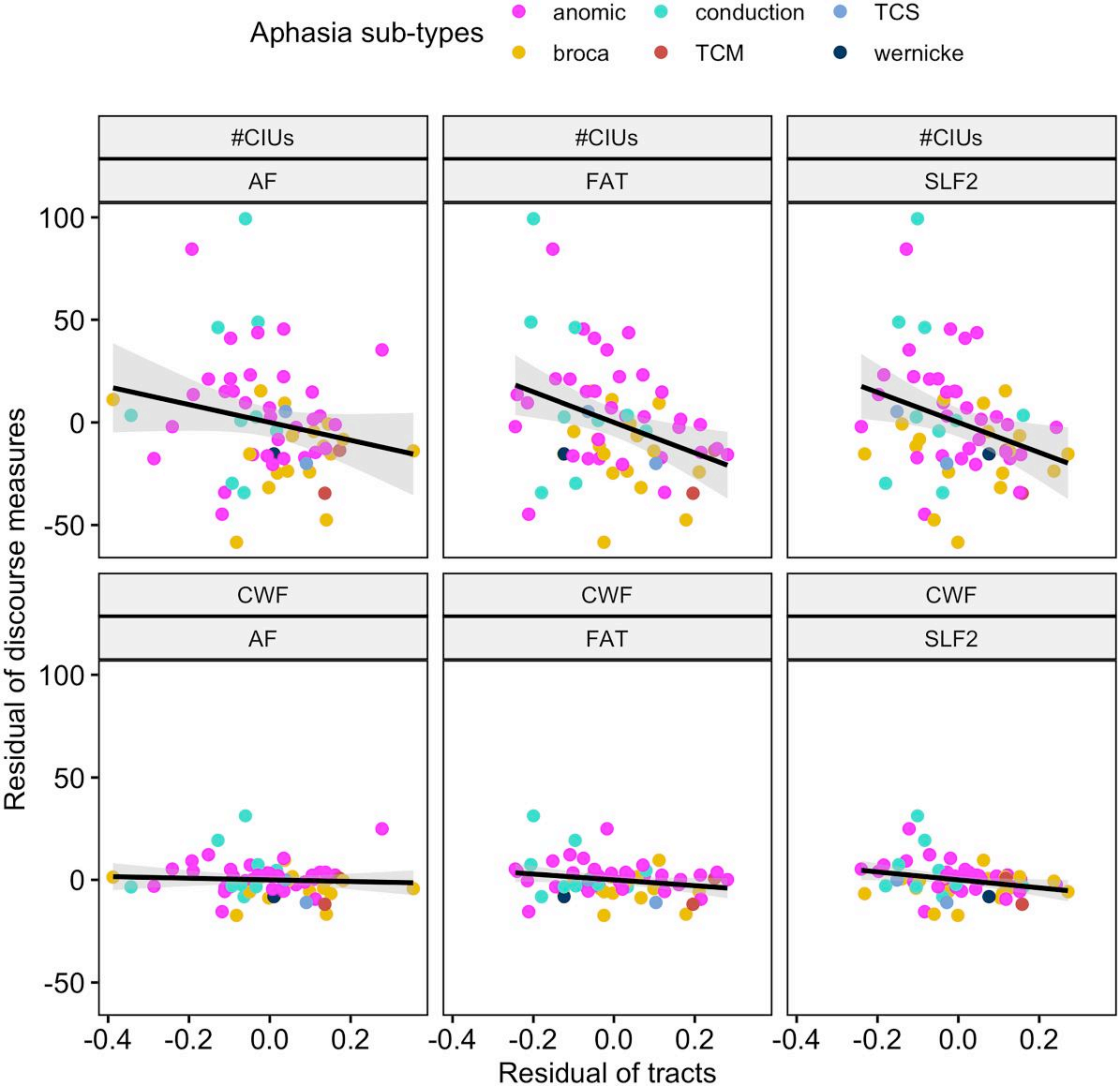
**Tract correlates of spoken discourse measures.** Scatterplots illustrating the partial correlations between tract damage percentage (*x*-axis) and discourse measures (*y*-axis), controlling for total lesion size. The analyses indicated significant correlation between both content word fluency (CWF) and correct information unit (#CIU) with the frontal aslant tract (FAT) (CWF: *r* = −0.22, *P* = 0.04; #CIU: *r* = −0.34, *P* = 0.003) and the second branch of superior longitudinal fasciculus (SLF) (CWF: *r* = −0.27, *P* = 0.02; #CIU: *r* = −0.30, *P* = 0.008). Each data point represents a subject (*N* = 63), including anomic aphasia (*n* = 31), Broca’s aphasia (*n* = 17), conduction aphasia (*n* = 9), transcortical motor aphasia (TCM) (*n* = 3), transcortical sensory aphasia (TCS) (*n* = 2) and Wernicke’s aphasia (*n* = 1). AF = arcuate fasciculus.

Tract LSM (Fig. 3) showed significant correlation between both CWF and #CIU with the FAT (CWF: *r* = −0.22, uncorrected *P* = 0.04; #CIU: *r* = −0.34, uncorrected *P* = 0.003) and the second branch of SLF (CWF: *r* = −0.27, uncorrected *P* = 0.02; #CIU: *r* = −0.30, uncorrected *P* = 0.008; Fig. 3). #CIU additionally correlated with the AF (CWF: *r* = −0.06, uncorrected *P* = 0.31; #CIU: *r* = −0.21, uncorrected *P* = 0.05). Other tracts did not significantly correlate with CWF or #CIU (CWF: *r* > −0.04, uncorrected *P* > 0.37; #CIU: *r* > −0.15, uncorrected *P* > 0.11).

## Discussion

CWF approach was recently developed to provide an accurate, efficient and clinically applicable measure of CWF during spoken discourse in PWA without the need to collect, transcribe or code discourse samples.^12,14^ CIU coding is a reliable measure of discourse informativeness that is frequently used in research settings.^4^ Coding for CIU requires time-consuming, specialized transcription and quantitative analysis of discourse samples, limiting the feasibility of their use in clinical settings or as an outcome measure in randomized control trials. It has been previously suggested that CWF approach can potentially be used as a shortcut to CIU coding.^12^ The present study provided further behavioural and psychometric validation of the CWF approach in an independent large dataset of PWA. The present study also systematically explored the lesion underpinnings of CWF during spoken discourse in a large sample of people with post-stroke aphasia.

### Further validation of the content word fluency approach

The present results replicate the findings of Alyahya *et al*.^12^ in an independent large sample of PWA. We found a significant, strong positive correlation between CWF scores and the number of CIUs, indicating strong convergent validity between these two approaches. This consistency across different datasets enhances the reliability and generalisability of the CWF approach as an efficient measure of fluent production of relevant information during spoken discourse in PWA. The IRT analysis indicated that differences between items on the checklist can be adequately described by a single latent dimension that could be interpreted as item difficulty. The adequacy of the Rasch model suggests that the items on the CWF checklist are homogeneously measuring a single underlying construct, which simplifies the interpretation of the results. This model also allows each item on the checklist to be ranked on a difficulty scale, which can be used to assess the functional range of the checklist.^34^ The items on the checklist were found to cover a fairly broad range of item difficulties, which makes the checklist sensitive to variations in performance across the entire spectrum of CWF within the sample.^35^ This distribution is crucial for the effectiveness of the checklist to accurately reflect both mild and severe deficits in CWF during spoken discourse. The coverage across a large functional range also suggests that the current number of words on the checklist is sufficient for capturing the variability in participants’ performance, providing comprehensive and reliable assessments without the need for additional items.^36^ Such validation is essential to ensure that CWF checklists can be broadly applied to various populations with aphasia in both clinical and research contexts.

The fluent production of content words during spoken discourse (measured using the CWF approach) was associated with disconnection of the left middle frontal gyrus, inferior parietal lobule and white matter tracts, including the superior longitudinal fasciculus, frontal aslant tract and superior thalamic radiations. These grey matter regions and white matter tracts overlap with the lesion correlates associated with deficits in discourse informativeness (measured using #CIU). Although there were small differences between CWF and #CIU results (#CIU was further associated with tract damage of the arcuate fasciculus), a formal direct comparison between the lesion maps of the two discourse approaches indicated that the two symptoms are adequately captured by a single map. These results are also very similar to the results of a prior lesion analysis of #CIU from 10 connected speech prompts,^6^ consistent with a strong relationship between the two approaches. Further neuroimaging studies on an independent sample are recommended, but the current evidence suggests that the transcription-less CWF approach can be used as a shortcut to #CIU coding.

### The lesion correlates of deficits in spoken discourse

Findings from the lesion-symptom-mapping analyses align with and extend the current understanding of the neural underpinnings involved in language processing, particularly discourse production. The involvement of the left middle frontal gyrus and inferior parietal lobule in CWF is consistent with previous studies that highlight the role of these regions in language production, particularly discourse informativeness,^9^ grammatical structure during narratives^21,37^ and discourse management,^38^ as well as executive functions.^39^ Furthermore, the left inferior parietal lobule has been implicated in semantic processing and was linked to systems involved in the perceptual processing of picture stimuli.^40^ The current findings also indicated that deficits in content word production during spoken discourse were associated with damage to dorsal white matter pathways, including frontal aslant tract, which connects the superior and inferior regions of the frontal lobe and supplementary motor areas, and the superior longitudinal fasciculus, which connects frontal, parietal and temporal regions. These tracts are integral to the dorsal language pathway, which is implicated in phonological and articulatory processing.^41^ These findings converge with prior evidence that damage to the frontal aslant tract is associated with reduced informativeness^6^ and fluency during spoken discourse.^9,42,43^ The superior longitudinal fasciculus is also involved in language motor planning and syntactic processing during language production.^44^ The identification of clusters in the superior thalamic radiations aligns with the role of thalamus, particularly through its connections with cortical regions via thalamic radiations, in attention and the integration of sensory and motor information during productive language execution.^45^ The identified neural network underlying the fluency of content word production during discourse suggests that these regions facilitate the integration of semantic, syntactic, phonological and executive information necessary for informative and fluent discourse production. Executive functions are essential for ensuring that spoken discourse conveys the intended relevant information of the topic under discussion.^2^ However, executive functions were not examined in this study, and thus, the presence or absence of executive impairments and their association with content word production during spoken discourse cannot be determined.

### Implications and future directions

CWF is a transcription-less approach developed to overcome challenges associated with the time-consuming and labour-intensive scoring of discourse responses.^12^ Using a pre-specified checklist, it provides an objective measure of the overall quantity of content words produced during spoken discourse that is nearly identical to a well-established, but laborious scoring system (i.e. #CIU). Compared to other discourse measures, the CWF checklist approach is much faster and easier to apply, even in real time during clinical examination,^14^ and does not rely on advanced linguistic skills, meaning it can be administered by healthcare professionals across disciplines (e.g. neurologists, psychiatrists and nurses) to screen for deficits in spoken discourse in aphasia. This efficient assessment of the informativeness and fluency of spoken discourse is useful for both clinical and research applications, for example, as part of a core set of discourse outcome measures.^46^

Core-lexicon is another transcription-less approach that has been developed to analyse discourse responses.^47,48^ Both core-lexicon and CWF use a checklist of words, but the lists of target words were developed differently and are scored differently. Core-lexicon assigns only one point to each lexical item, regardless of how often the word was produced, whereas CWF assigns a point each time a word from the checklist is produced, in any inflection form, and including when the word is used in different sentences. Furthermore, CWF gives points for synonyms, which is not permitted in Core-lexicon.^49^ While core-lexicon measures word retrieval,^47,48^ CWF assesses the fluency of content words during spoken discourse,^12,14^ although it is not a measure of speech fluency. It may be useful for future research to examine the relative merits of these two approaches and to compare CWF approach to core-lexicon on the same sample.

CWF checklists were developed for the BDAE ‘Cookie Theft’ picture description task, the ‘dinner party’ storytelling narrative, based on the discourse responses of non-brain-damaged adults.^12^ The BDAE version of the ‘Cookie Theft’ picture description task is widely used and forms a useful starting point, but this approach can be readily extended to other discourse elicitation tasks and stimuli, such as procedural discourse, the Modern Cookie Theft picture^50^ and the Western Aphasia Battery ‘Picnic Scene’ complex picture description task.^15^ The CWF approach has also been extended to other languages—an important gap in aphasia research,^51,52^ including Arabic using the ‘Kitchen’ storyboard and the ‘Lounge’ composite picture description.^14,53^ Continuing to refine this tool and examining its application with less constrained tasks, such as expository discourse, is vital for advancing its utility as an efficient assessment and outcome measure in aphasia.

The lesion analysis results, combined with other work on the lesion correlates of spoken discourse,^7-10^ indicate that disruptions of frontal-parietal connectivity play a particularly important role in discourse production deficits. This may be useful for prognosis, for selecting treatment strategies and for evaluating the effectiveness of treatments. As an efficient and easy-to-use measure of discourse content, CWF can play a crucial role in this form of clinical research and assessment.

## Conclusion

The present study used a large dataset of discourse responses from PWA and state-of-the-art LSM analysis methods to further validate the CWF approach and identify the lesion correlates associated with impaired CWF during spoken discourse. The evidence confirms the reliability and robustness of CWF as a transcription-less, time-efficient and clinically applicable approach to assessing the fluency and informativeness of spoken discourse using target checklists. This approach allows discourse responses to be collected and scored in real time during clinical and research assessments, eliminating the need for offline transcription and quantitative analysis. The neuroimaging findings provide strong evidence that, in chronic post-stroke aphasia, deficits in producing content words during spoken discourse are associated with disrupted connectivity of left frontal and parietal regions and their white matter pathways. These insights have potential clinical implications, from improving targeted rehabilitation strategies to predicting recovery outcomes.

## Data Availability

The data and codes used in this study are available on our OSF project page (https://osf.io/rhmqv/). The CWF checklist is available in the appendix of our previous publication.^12^
